# Upregulation of OTUD7B (Cezanne) Promotes Tumor Progression via AKT/VEGF Pathway in Lung Squamous Carcinoma and Adenocarcinoma

**DOI:** 10.3389/fonc.2019.00862

**Published:** 2019-09-11

**Authors:** Dan-dan Lin, Yang Shen, Shu Qiao, Wen-wen Liu, Lishuang Zheng, Ya-nan Wang, Naipeng Cui, Yun-fan Wang, Shuli Zhao, Jian-hong Shi

**Affiliations:** ^1^Central Laboratory, Hebei Key Laboratory of Cancer Radiotherapy and Chemotherapy, Affiliated Hospital of Hebei University, Baoding, China; ^2^Department of Breast Surgery, Affiliated Hospital of Hebei University, Baoding, China; ^3^Department of Pathology, Affiliated Hospital of Hebei University, Baoding, China; ^4^General Clinical Research Center, Nanjing First Hospital, Nanjing Medical University, Nanjing, China

**Keywords:** OTUD7B, lung squamous carcinoma, lung adenocarcinoma, prognosis, VEGF, Akt

## Abstract

OTUD7B, a multifunctional deubiquitinylase, plays an essential role in inflammation and proliferation signals. However, its function in lung cancer remains largely unknown. The aim of this study was to evaluate the prognostic significance of OTUD7B in patients with lung adenocarcinoma and squamous carcinoma and to characterize its molecular mechanisms in lung cancer progression and metastasis. Two tissue microarrays containing 150 pairs of lung squamous carcinoma and matched adjacent non-cancer tissues, and one tissue microarray containing 75 pairs of lung adenocarcinoma and adjacent non-cancer tissues were included, and immunohistochemical staining was performed to assess the clinical relevance of OTUD7B in non-small cell lung cancer. OTUD7B is highly expressed in both lung squamous carcinoma and adenocarcinoma and correlates with a worse prognosis. MTT proliferation, colony formation, migration and invasion assays and immunoblotting assay in NCI-H358 and A549 cell lines suggested that OTUD7B enhances EGF-induced Akt signal transduction and promotes lung cancer cell proliferation and migration. Immunohistochemical staining of large-scale lung cancer subjects (171 cases) revealed positive correlation of OTUD7B and VEGF expression. ELISA and tube formation assay revealed OTUD7B promotes VEGF production and angiogenesis. NCI-H358 tumor model demonstrated OTUD7B is required for lung tumor progression by facilitating activation of Akt signaling. These findings collectively identified OTUD7B as an independent predictive factor for the prognosis of non-small cell lung cancer and revealed OTUD7B promotes lung cancer cell proliferation and metastasis via Akt/VEGF signal pathway.

## Introduction

Lung cancer is one of the most common cancers and leading causes of cancer-related deaths worldwide. Most cases of lung cancer are non-small cell lung cancer (NSCLC). NSCLC can be further sorted into several types, including lung squamous carcinoma (LUSC) and lung adenocarcinoma (LAD), which are the most commonly diagnosed histological subtypes of NSCLC (1). High incidence of post-chemotherapy metastasis and recurrence generates challenge as for its resistance to conventional systemic radiotherapy and chemotherapy (2). The importance of understanding the molecular biology of LUSC and LAD has recently gained considerable attention.

Ubiquitination is an important mechanism that regulates cancer progression (3, 4). Deubiquitylases (DUBs) are enzymes that hydrolysis ubiquitin chains from target proteins and contribute to their stabilization and activation (5, 6). DUBs play essential roles in inflammation and proliferation signal transduction and are considered effective therapeutic targets against lung cancer. OTUD7B, a DUB belonging to A20 subgroup of ovarian tumor (OUT) protein superfamily, functions as an inflammation and NF-κB signaling regulator (7–11). Accumulate data suggested that OTUB7B appears to be the primary regulatory mechanism to growth signals, including activation of mTORC2/Akt pathway (12), regulation of E2F1-dependent HIF2α expression (13), stabilization of HIF1α (14), and EGFR (15).

Although some evidence revealed the role of OTUD7B in cancer progression (10, 12, 15–18), the molecular mechanisms of OTUD7B participation in invasion and metastasis in NSCLCs remains elusive. Here, we demonstrate that OTUD7B were predominantly upregulated in LUSC and LAD tissues compared with adjacent normal lung tissues. OTUD7B upregulation predicts an increased risk for cancer metastasis in LUSC and LAD patients. Moreover, we found that OTUD7B regulation in lung cancer progression associates with tumor angiogenesis via Akt/VEGF pathway.

## Materials and Methods

### Antibodies and Reagents

Antibodies for OTUD7B (16605-1-AP) and JNK (10023-1-AP) were from Proteintech Group, Inc. (Chicago, USA). Antibody for GAPDH (AC033) was from Abclonal (Wuhan, China). Antibodies for p70S6K (2708), p-p70S6K T389 (9206), p-p70S6K T421/S424 (9204), ERK1/2 (9102), p-ERK1/2 T202/Y204 (9101), p-Akt S473 (4060), p-p38 T180/Y182 (9215), and p-JNK T183/Y185 (4668) were purchased from Cell Signaling Technology (Boston, USA). Antibody for p38 (35478) was from SAB. Antibody for Akt1(SC-5298) was from Santa Cruz Biotechnology (Dallas, USA). Human VEGFA monoclonal antibody (M808) and Biotin-labeled VEGFA polyclonal antibody were purchased from Thermo Fisher Scientific (Waltham, USA). EGF was from Peprotech (Suzhou, China).

### Plasmids and shRNAs

The human OTUD7B cDNA (accession NM_020205.4) was cloned into the lentivirus vector pLenti-CMV-EGFP-3Flag to create the GFP or GFP-OTUD7B expression vector, and the OTUD7B cDNA sequence was confirmed by sequencing. LV3 lentiviral vectors encoding shRNAs silencing OTUD7B or a non-silencing control shRNA were purchased from GenePharma (Suzhou, China). The sequences of OTUD7B shRNAs: shOTUD7B#1: TTCTCCGAACGTGTCACGT; shOTUD7B#2: GCTGCGGAAAGCTTTGTATGC.

### Tissue Array and Immunohistochemical Staining

Three tissue microarrays containing 225 pairs of lung cancer and corresponding non-cancer tissues with survival times were purchased from BioChip (Shanghai, China), of which 150 cases are LUSC and 75 cases are LAD. These samples were collected from 2004 to 2009. The study was approved by Institutional Review Board of Hebei University Affiliated Hospital. All of the patients provide informed consent. The immunohistochemistry assay and the analysis of clinicopathological features were based on 75 cases of LAD and 150 cases of LUSC from tissue microarray, excluded a few cases because of missing data. Detailed clinical and pathologic information of patients are displayed in Table 1.

**Table 1:** Patient characteristics.

**Variable**	**No. of patients (%)**
No. of NSCLC patients	225 (100)
No. of LUSC patients	150 (66.7)
No. of LAD patients	75 (33.3)
Age: Median [range]	62 [25–84]
Gender	
Female	46 (20.4)
Male	179 (79.6)
AJCC stage	
I	81 (45)
II	54 (30)
III	44 (24.4)
IV	1 (0.6)
Lymphatic metastasis	
Absent	122 (56.5)
Present	94 (43.5)
Distant metastasis	
Absent	224 (99.6)
Present	1 (0.4)
Prognosis	
Survival	145 (64.4)
Death	80 (35.6)

We performed immunohistochemical (IHC) staining for OTUD7B and VEGF on the same paraffin embedded tissue blocks that were used for clinical diagnosis. Immunohistochemistry was performed using the avidin–biotin complex method, including heat-induced antigen-retrieval procedures. Incubation with polyclonal antibodies against OTUD7B (16605-1-AP; 1:1,000 dilution; Proteintech), VEGF (19003-1-AP; 1:500 dilution; Proteintech) was performed at 4°C overnight followed by incubation with horseradish-peroxidase (HRP)-conjungated secondary antibody kit. Staining was assessed by pathologists who were blinded to the sample origins and the patient outcomes. Each sample was assigned a score according to the intensity of the nucleic, cytoplasmic and membrane staining (no staining = 0; weak staining = 1, moderate staining = 2, and strong staining = 3) and the extent of stained cells (0–4 = 0, 5–25 = 1, 26–50 = 2, 51–75 = 3, and 76–100 = 4). Immunostaining score formula = score of positive cell percentage × score of staining intensity. OTUD7B expression was qualified as low (IHC score 0–3), medium (IHC score 4–7), and high (IHC score 8–12).

### Gene Expression Databases

Gene expression data were downloaded from public databases including Gene Expression Omnibus (GEO) and The Cancer Genome Atlas (TCGA). The cBioPortal for Cancer Genomics system (www.cbioportal.org) was used to analyze OTUD7B gene. R2: Genomics Analysis and Visualization Platform system (http://r2.amc.nl) was used to analyze the correlation between OTUD7B and VEGF.

### Cell Culture, Gene Silencing, and Overexpression

Human NSCLC cell lines NCI-H358 and A549, human embryo kidney (HEK) 293 cell line and human endothelial EA.hy926 cell line were obtained from Cell Resource Center of Shanghai Institutes for Biological Sciences, Chinese Academy of Science, China. NCI-H358 and A549 cells were cultured in RPMI-1640 supplemented with 10% FBS. HEK293 and EA.hy926 cells were maintained in Dulbecco's Modified Eagle's Medium with 10% FBS. All cell lines were cultured in a humidified atmosphere with 5% CO_2_ at 37°C.

For gene silencing, lentiviral particles were prepared by transfecting HEK293 cells with LV3 lentiviral vectors encoding specific shRNAs or control shRNAs along with packaging plasmids. The packaged viruses were then used to infect the indicated cells, followed by selection of the infected cells by puromycin (LV3 vector carries the puromycin resistance gene) to establish stable cell lines. Immunoblotting assays were performed to examine the silencing efficiency. For overexpression studies, lentiviral transduction was performed as described above.

### Immunoblot Assay

Total cell lysates and tumor tissue extracts were prepared and subjected to immunoblot assay as described previously (19). Briefly, crude proteins were extracted, resolved by SDS-polyacrylamide gel electrophoresis and transferred onto a PVDF membrane. Membrane were then blocked with 5% non-fat milk in TBST buffer for 2 h at 37°C and incubated with primary antibodies overnight at 4°C. After incubation with the appropriate secondary antibody, the immunoblot signal was visualized using ECL reagent.

### Cell Proliferation and Colony Formation Assays

Cell proliferation was assessed by MTT assay. NCI-H358 cells were seeded into 96-well plates at a density of 1 × 10^4^ cells/well in 0.1 mL medium and cultured for different periods of time as indicated. Then, 20 μL MTT reagent (0.5 mg/mL in PBS) was added to each well and the plate was incubated for 2 h before detection. The purple-blue MTT formazan precipitate was dissolved in 100 μL DMSO. The absorbance was measured at 490 nm using a BioTek Epoch Spectrophomometer (BioTek, Winooski, USA). For colony formation, NCI-H358 cells were harvested and pipetted well to become single-cell suspension in RPMI-1640 with 10% FBS at a concentration of 750 cells/mL. Cells were then planted into a 6-well plate and incubated at 37°C in a humidified 5% CO_2_ atmosphere for 2 weeks. After incubation, the cells were fixed with methanol and stained with hexamethyl pararosaniline. Colony formation was then photographed and counted under a phase-contrast microscope.

### Cell Migration and Invasion Assays

Cell migration assay was performed according to the previously described protocol (20). Cells were grown to 100% confluence on glass slides and a 3-mm-wide scratch was made using a cell scraper. Slides were then incubated at 37°C for 24 h in DMEM containing 10 g/L BSA and 1% FBS. After incubation, the cells were fixed with methanol and stained with hexamethyl pararosaniline. The “wound” was observed under a light microscope. Each sample was randomly selected five fields and photographed. Cell migration was expressed as the migrated distance. For cell invasion assay, a modified Boyden transwell chamber with 8.0 mm pores was used. The transwell chamber was pre-coated with 100 μL of Matrigel (10 mg/mL) overnight at 4°C. Cells were seeded (10^5^ cells per well) in the upper chamber of the transwell system in DMEM with 0.5%FBS, and DMEM with 10% FBS were added to the lower chamber of the transwell system at 500 μL per well. Then, cells were incubated at 37°C for 6 h. Cells remaining on the upper membrane surface were mechanically removed, and the cells that invaded and migrated to the lower surface of the filter were fixed and stained with 0.5% crystal violet and observed under the microscope. Each sample was randomly selected five fields and the cells that moved to the lower surface of the filter were photographed and counted.

### VEGF ELISA and Tube Formation Assay for Angiogenesis

NCI-H358 cells were seeded and incubated for 24 h in culture media. Then, media was removed and cells were incubated in serum-free RPMI-1640 for additional 48 h. Then, conditioned media were obtained. Vascular endothelial growth factor (VEGF) in conditioned media was quantified using VEGF ELISA Kits (Neobioscience Technology, China). To investigate the effect of the conditioned media on the angiogenic activity of EA.hy926 cells *in vitro*, a tube formation assay was performed according to the protocol described previously (21). EA.hy926 cells were seeded into a 24-well plate pre-coated with 100 μL of 10 mg/mL chilled Matrigel (Corning, Bedford, MA) at a density of 1 × 10^5^ cells/well in 0.5 mL of culture medium. After 8 h of incubation, the tube-like structures in five randomly selected microscopic fields in each well were imaged using phase-contrast microscopy and the total lengths were quantified using Image-Pro Plus software 6.0 (Media Cybernetics, Inc., Rockville, MD).

### Tumor Xenograft

Male BALB/c-nu mice, 4–5 weeks old, were purchased from the Beijing HFK Bioscience Co., Ltd. (Beijing, China). All of the mice were bred and housed in a specific pathogen-free environment at Hebei University Laboratory Animal Research Center. All experiments were approved by the Animal Research Ethics Committee of the authors' institution. NCI-H358 cells were cultured in RPMI-1640 supplemented with 10% FBS. Nude mice were injected subcutaneously with a cell suspension of 0.1 ml in PBS containing 1 × 10^6^ tumor cells into the nude mice. Each experimental group consisted of 6 mice. The challenged mice were monitored for tumor growth, and tumor size was expressed as tumor volume. The volume of the tumors was estimated according the formula: Volume = ½ × *a* × *b*^2^, where *a* and *b* represent the largest and smallest diameters, respectively. Mice were sacrificed at day 15 after injection. The xenograft tumors were dissected, weighted, and photographed.

### Statistical Analysis

Statistical analysis was performed using the SPSS software package (version 19.0, USA). Two-tailed unpaired or paired Student's *t*-tests or one-way analysis of ovariance (ANOVA) with Tukey's multiple comparisons were used according to the number of groups compared. Survival curves were generated using the Kaplan–Meier method, and differences between curves were estimated by the two-sided log-rank test. *P*-values < 0.05 were considered significant and the level of significance expressed as follows: ^*^P < 0.05; ^**^P < 0.01; ^***^P < 0.001; ^****^P < 0.0001.

## Results

### Elevated OTUD7B Expression Correlates With LUSC and LAD Progression

**Figure 1:**
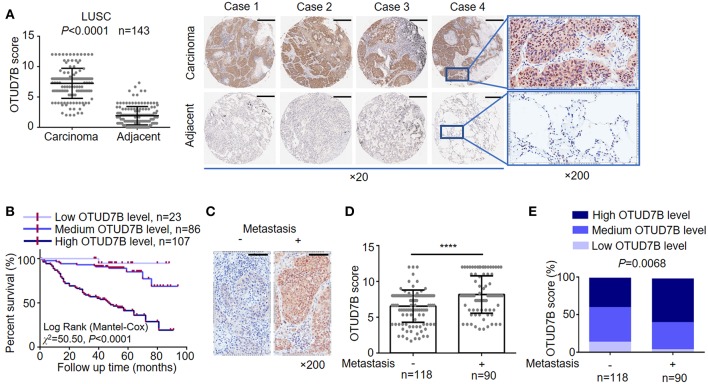
OTUD7B is highly expressed in NSCLCs and correlated with a worse prognosis. **(A)** OTUD7B expression scores in LUSC and adjacent non-cancer tissues are shown as scatter dot plots. Small horizontal bars indicate the mean ± s.d. Each dot represents an individual sample. LUSC tissues were compared with matched adjacent non-cancer tissues using paired *t*-test. Right, Representative images of IHC staining of OTUD7B expression from 4 cases are shown. Magnification, ×20 (middle) and ×200 (right); scale bars, 400 μm. **(B)** Kaplan-Meier survival curve of high, medium and low OTUD7B level NSCLC. Marks on graph lines represent censored samples. *P*-value refers to two-sided log-rank tests. **(C)** Representative images from IHC staining of OTUD7B expression in tumors with or without metastasis (lymph node metastasis and distant metastasis) are shown. Magnification, ×200; scale bars, 100 μm. **(D)** Scatter dot plots of OTUD7B expression in the two groups of subjects described in **(C)** are shown. Data were analyzed using unpaired *t*-test and are shown as mean ± s.d. ****P < 0.0001. **(E)** The percentage of tumors in the two groups of subjects described in **(C)**. Data were analyzed using Pearson's χ^2^ test.

OTUD7B, also called Cezanne, was identified as an essential regulator of the NF-κB pathway and cancer proliferation, although its physiological function in lung cancer has not been well-defined (10, 11, 22). The OTUD7B gene was frequently amplified in NSCLC, including LUSC and LAD, according to the TCGA DNA sequencing results (Supplementary Figure S1). To investigate the potential clinical relevance of OTUD7B in lung cancer, we assessed lung cancer tissue samples and matched adjacent normal lung tissue samples from 214 human subjects (143 LUSC cases and 71 LAD cases). IHC analysis revealed that OTUD7B expression was significantly upregulated in both LUSC (Figure 1A) and LAD (Supplementary Figures S2A–D) tissues compared with adjacent tissues. To better understand the relevance of OTUD7B to cancer a follow-up analysis of patient survival was performed, and the result demonstrated that patients whose tumor had high OTUD7B levels had significantly poorer survival than those with low OTUD7B levels (Figure 1B). Next, we divided the samples into groups based on metastasis (Figures 1C–E) and the AJCC stage (Supplementary Figures S3A–C) to study the correlation of OTUD7B expression with lung cancer development. OTUD7B expression was higher in tumors with lymph node or distance metastasis than that without metastasis (Figures 1C–E). OTUD7B expression was also lower in low-AJCC stage (AJCC stage I and II) and higher in high-TNM stage (TNM stage III and IV) (Supplementary Figures S3A–C). These data indicate that OTUD7B promotes the metastasis and progression in LUSC and LAD.

### OTUD7B Is Associated With Cellular Proliferation, Migration, and Colony Formation in NSCLC Cell Lines

**Figure 2:**
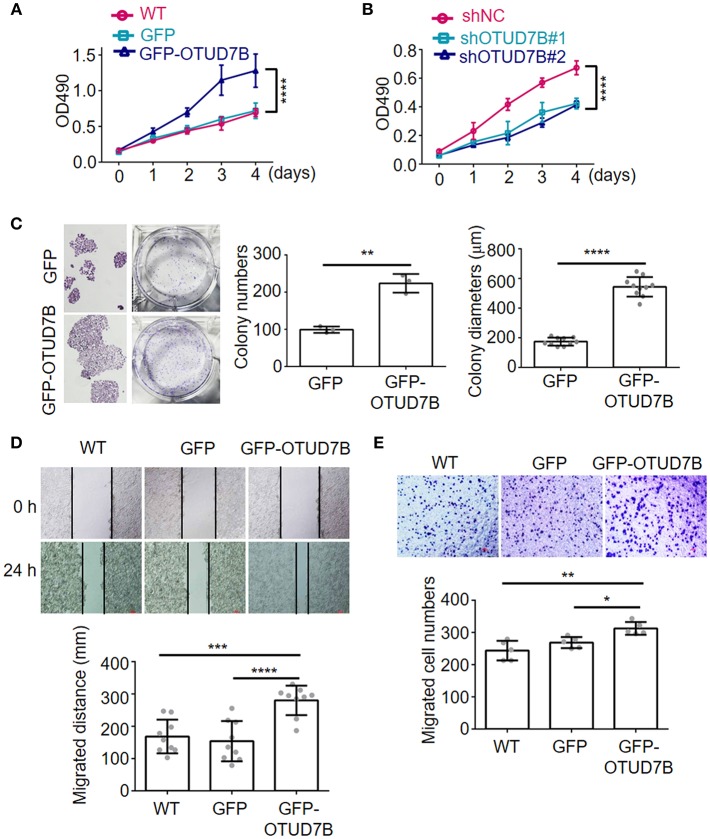
OTUD7B promotes proliferation, migration and colony formation in lung cancer cells. **(A,B)** NCI-H358 lung cancer cells were transduced with GFP or GFP-OTUD7B (A) or transduced with a non-targeting control shRNA (shNC) or two different OTUD7B-specific shRNAs (B). Cells proliferation was tested using MTT assay and the absorbance was measured at 490 nm. **(C)** NCI-H358 cells were transduced with GFP or GFP-OTUD7B. Cell proliferation were determined by colony formation analysis. **(D,E)** Wound healing assay **(D)** and transwell invasion assay using Boyden chamber **(E)** in NCI-H358 cells were performed and photographed under a light microscope (magnification, ×100). Representative images are shown (upper panels). Data are shown as mean ± s.d. *P < 0.05, **P < 0.01, ***P < 0.001, ****P < 0.0001.

To address the consequences of OTUD7B-mediated NSCLC progression, we established NSCLC cell line NCI-H358 stably overexpressing OTUD7B (Supplementary Figure S4A) and performed MTT cell proliferation assay. As expected, overexpression of OTUD7B resulted in an increased proliferation activity in NCI-H358 cells (Figure 2A). Transducing specific shRNA targeting OTUD7B into NCI-H358 cells further confirmed that knockdown of endogenous OTUD7B correlates with decreased cell growth (Figure 2B, Supplementary Figure S4B). Furthermore, we examined the effect of OTUD7B on NCI-H358 cell proliferation using a colony formation assay. As shown in Figure 2C, both the colony numbers and colony diameters increased significantly in OTUD7B-overexpressed cells. When endogenous OTUD7B was knocked down, the number and diameter of colony decreased significantly compared with wild type and GFP-overexpression groups (Supplementary Figures S5A–C).

To further investigate the roles of OTUD7B in NSCLC cell biology, cell migration and invasion assays were performed in NCI-H358 and A549 cells. Wound-healing cell migration assay indicated that OTUD7B overexpression significantly promoted cell migration (Figure 2D, Supplementary Figure S6A). Matrigel pre-coated Boyden chamber was used to analyze cell invasion in OTUD7B-overexpressing and control cells. Cells that migrated through the filter were stained and photographed and the results showed that migrated cell numbers significantly increased in OTUD7B-overexpressing cells (Figure 2E, Supplementary Figure S6B).

### OTUD7B Promotes p-Akt Level in NSCLC Cell Lines

**Figure 3:**
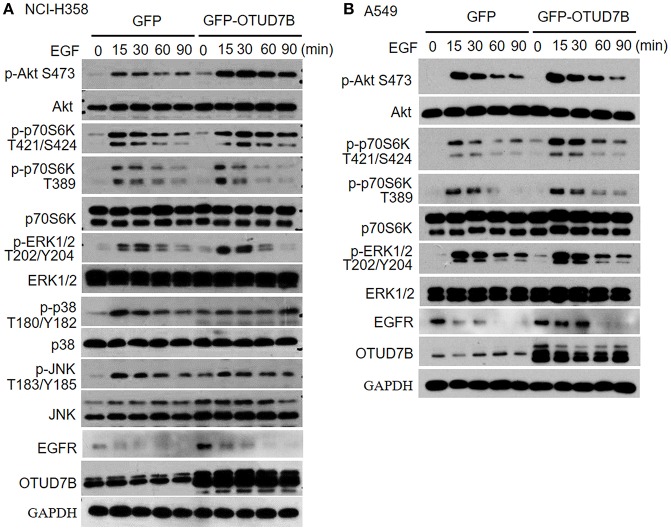
OTUD7B activates Akt and ERK1/2 signals in NSCLC cell lines. NCI-H358 cells **(A)** or A549 cells **(B)** transduced with GFP or GFP-OTUD7B were stimulated with EGF (500 ng/ml) for indicated times and total cell lysates were harvest and subjected to immunoblotting assay.

Because Akt, ERK1/2, p38, and JNK signaling pathways is crucial for many fundamental cellular processes, we sought to determine whether OTUD7B was involved in these signaling pathways in NCI-H358 and A549 NSCLC cell lines. NCI-H358 cells were stably transduced with GFP or GFP-OTUD7B. Cells were treated with 500 ng/mL of EGF for 15, 30, 60, and 90 min as indicated and immunoblotting was performed to examine phosphorylated Akt, ERK1/2, p38, and JNK levels, as well as an Akt downstream kinase, p70S6K pohsphorylation in GFP- and GFP-OTUD7B-overexpressing cells. OTUD7B overexpression markedly increased phosphor-Akt and phospho-p70S6K levels in EGF stimulated NCI-H358 cells (Figure 3A). A slight elevation of phospho-ERK1/2 expression was also detected in OTUD7B-overexpressed NIC-H358 cells (Figure 3A). Moreover, OTUD7B overexpression also facilitated phospho-Akt, ERK1/2, and p70S6K levels in another NSCLC cell line A549 (Figure 3B, Supplementary Figure S4C). EGFR is an important receptor which is well-known as target for personalized therapy (23). We also detected EGFR protein levels in EGF-stimulated GFP- and GFP-OTUD7B-overexpressing cells. As shown in Figures 3A,B, compared with control cells EGFR protein level was significantly higher in OTUD7B-overexpressing cells. Moreover, degradation of EGFR in EGF-stimulated cells was much more rapid in control cells than that in OTUD7B-overexpressing cells. These results indicated that OTUD7B promotes NSCLC cell proliferation through activating EGFR, Akt and ERK1/2 pathways.

### VEGF Correlates With OTUD7B Expression in LUSC and LAD

**Figure 4:**
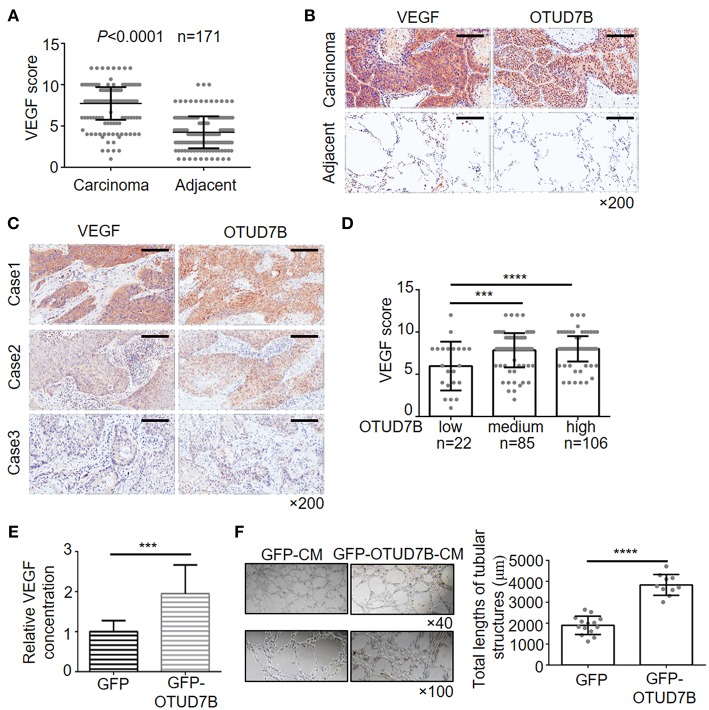
Positive correlation of OTUD7B with VEGF expression and its role in VEGF production and angiogenesis. **(A)** The expression scores of VEGF (*n* = 171) were compared between NSCLC tumors and matched adjacent normal tissue using paired *t-*test. Data are shown as mean ± s.d. **(B)** Representative images from IHC staining of VEGF in the same NSCLC tumor. Magnification, ×200; Scale bars, 100 μm. **(C)** Representative images from IHC staining of VEGF and OTUD7B expression in two serial sections of the same tumor from three cases. Magnification, ×200; Scale bars, 100 μm. **(D)** Scatter dot plots of VEGF expression in NSCLC from 213 subjects. The subjects were divided into three groups based on OTUD7B expression scores in the tumors, representing low, medium and high expression of OTUD7B. Data were analyzed by one-way ANOVA and Tukey's multiple comparisons test. **(E)** Media from GFP- or GFP-OTUD7B-overexpressing NCI-H358 cells was removed, washed in RPMI-1640 with 0.5% FBS and incubated for an additional 24 h in RPMI-1640 with 0.5% FBS. GFP-conditioned media (GFPCM) and GFP-OTUD7B-conditioned media (GFP-OTUD7B-CM) were collected, and VEGF expression were analyzed by ELISA. Absorbance was measured at 450 nm. **(F)** EA.hy926 endothelial cells were pretreated with GFP-CM and GFP-OTUD7B-CM for 24 h. Subsequently, pretreated EA.hy926 cells were seeded on Matrigel for 8 h to observe tube formation. Representative photographs are shown (left). Tube lengths were quantitated using IMAGE-PRO PLUS software (*n* = 5 per group). Data are shown as mean ± s.d. ***P < 0.001, ****P < 0.0001.

Recent data indicated that Akt is an important signaling regulator of VEGF production and tumor angiogenesis (24–26). Previous research has also reported that OTUD7B regulates hypoxia inducible factors 1α and 2α protein (13, 14), which are known effective regulators of VEGF and angiogenesis in cancer. However, little is known about the relation of OTUD7B and VEGF. First, we analyzed the correlation of OTUD7B and VEGF using R2: Genomics Analysis and Visualization Platform (http://r2.amc.nl). As shown in Supplementary Figure S7, OTUD7B expression was positively associated with VEGF expression (*r* = 0.239, *p* = 1.00e-06). To further determine their relationship, we next examined the expression of VEGF in NSCLC carcinoma tissues and matched normal lung tissues using IHC staining. An analysis of large-scale subjects (171 cases, including 116 cases of LUSC and 55 cases of LAD) demonstrated that VEGF were significantly upregulated in NSCLC carcinoma tissues compared with matched adjacent normal tissues (Figures 4A,B). Importantly, we observed a positive correlation between OTUD7B and VEGF levels among the examined subjects (Figures 4C,D).

To confirm the relevance between OTUD7B and VEGF, we further examined the VEGF concentration in conditional media of NSCLC cell lines that stably overexpress or knockdown OTUD7B. Conditioned media (CM) from NCI-H358 cells stably expressing control-GFP (GFP-CM) or GFP-OTUD7B (GFP-OTUD7B-CM) were collected and secreted VEGF levels were examined using ELISA assay. Overexpression of OTUD7B results in higher VEGF secretion in NCI-H358 cells (Figure 4E). When knockdown of endogenous OTUD7B, secreted VEGF levels decreased (Supplementary Figure S8A). Moreover, we examined EGF (another growth factor) secretion in OTUD7B overexpress or knockdown cells. Compared to the control group, there was no significant change of EGF secretion from OTUD7B overexpressing or knockdown NCI-H358 cells (Supplementary Figures S8B,C).

VEGF is an important factor involved in angiogenesis, resulting in tumor progression. Endothelial cells proliferation, migration and tube formation are key processes of angiogenesis which are stimulated by tumor-secreted pro-angiogenic factors, such as VEGF (27, 28). We next analyzed whether OTUD7B-induced VEGF secretion from NSCLC cells affect endothelial cell function and promotes angiogenesis. GFP-CM and GFP-OTUD7B-CM from NCI-H358 cells were collected and EA.hy926 endothelial cells were pretreated with GFP-CM and GFP-OTUD7B-CM for 24 h, and then seeded on Matrigel to determine tube formation. As shown in Figure 4F, tube length in GFP-OTUD7B-CM-treated EA.hy926 cells was significantly longer than GFP-CM-treated cells. Moreover, CM from control (shNC-CM) and OTUD7B-knockdown NCI-H358 cells (shOTUD7B#1-CM and shOTUD7B#2-CM) were collected and CM-induced tube formation analysis showed that tube length in shOTUD7Bs (shOTUD7B#1 and shOTUD7B#2)-CM-treated EA.hy926 cells was significantly shorter than shNC-CM-treated cells (Supplementary Figure S8D). Moreover, VEGFR2, an important VEGF receptor, was detected in EA.hy926 endothelial cells and lung cancer cells. The results demonstrated a higher expression of VEGFR2 in ehdothelial cells whereas VEGFR2 levels could hardly be detected in NSCLC cells (Supplementary Figures S9A,B). These results suggest that OTUD7B promotes VEGF production and NSCLC angiogenesis *in vitro*.

### OTUD7B Facilitate Akt Phosphorylation and NSCLC Tumorigenicity *in vivo*

**Figure 5:**
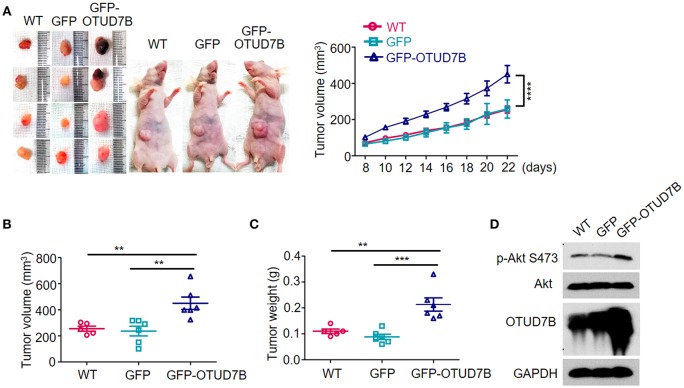
OTUD7B promotes *in vivo* tumorigenicity of NSCLC via Akt signaling activation. **(A)** Nude mice were injected s.c. with wild type (WT), GFP- or GFP-OTUD7B-overexpressing NCI-H358 cells at 5 × 10^6^ cells per site. Tumor volume was monitored every 2 days after subcutaneous injection and tumor growth curve is shown (right). Tumor volume was calculated by the formula: V = ½ × *a* (length) × *b*^2^ (width). Tumors were harvested from the mice 22 days after subcutaneous injection and photographed (left). **(B,C)** The volume **(B)** and the weight **(C)** of the harvested tumors was measured. **(D)** Total lysates of tumor tissues were subjected to immunoblotting using anti-p-Akt and anti-Akt antibodies. Data are shown as mean ± s.e.m. **P < 0.01, ***P < 0.001.

The NCI-H358 tumor bearing mouse model was established to further examine the tumorigenic role of OTUD7B. Wild type GFP- or GFP-OTUD7B overexpressing NCI-H358 cells were subcutaneously injected s.c. into the nude mice. The challenged mice were monitored for tumor growth. OTUD7B overexpression dramatically promoted tumorigenicity and tumor growth speed in nude mice (Figures 5A–C). Furthermore, Akt phosphorylation at S473 site was increased in OTUD7B-overexpressing tumors compared to the wild type and GFP control tumors (Figure 5D). To further confirm the role of OTUD7B in lung cancer progression, OTUD7B stably knockdown NCI-H358 cells were used to generate xenograft nude mouse model. Knockdown of OTUD7B in NCI-H358 cells significantly decreased Akt phosphorylation at S473 and inhibited tumor growth (Supplementary Figures S10A–D). These results suggest that OTUD7B is required for lung tumor progression at least partially by facilitating activation of Akt signaling.

## Discussion

Lung and bronchus cancer continues to be the leading cause of cancer mortality worldwide in both males (76,650 estimated deaths in 2019) and females (66,020 deaths) for more than 25 years (29). In spite of the rapid development of diagnostic and therapeutic technologies for NSCLC, its outcome remains poor, which may due to the lack of specific targets at the early stage (2, 30). Recent studies have demonstrated OTUD7B as a gene highly expressed in lung adenocarcinoma tissues (12, 18). In the present study, OTUD7B was revealed to overexpressed within LAD and LUSC tissues compared with adjacent noncancerous tissues. High expression of OTUD7B predicts metastasis and poor survival in NSCLC patients, which suggested potential role of OTUD7B in lung cancer initiation and progression.

In recent years, increasing evidence indicated the vital role of ubiquitinases and DUBs in the progression of NSCLC (3, 4, 31, 32). OTUD7B acts as a multifunctional DUB by targeting Lys11- (33, 34), Lys48- (8, 9), and Lys63-linked (12) ubiquitin chains. The essential role of OTUD7B in the activation of inflammation (7, 9, 35) and NF-κB pathway (8, 11) has been extensively investigated. However, the role of OTUD7B in tumor growth and metastasis signals is still not clear. We demonstrated OTUD7B expression level affects cell growth and colony formation in NCI-H358 cells. Phosphorylation of Akt, as well its downstream kinase, p70S6K, and ERK1/2 was significantly elevated by OTUD7B overexpression in EGF-treated NSCLC cell lines, NCI-H358 and A549. These data suggested that OTUD7B functions as an oncogene via facilitating Akt activation.

Angiogenesis is the formation of new blood vessels from pre-existing vasculature, which is essential for solid tumor growth and metastasis. The VEGF signal pathway has been well-characterized in lung cancer pathogenesis and anti-VEGF treatment has been considered one of the most effective strategies against advanced NSCLC. Recent evidence demonstrated Akt participates in VEGF production (24, 36). OTUD7B promotes EGF-stimulated Akt activation in NSCLC cell lines, however the correlation of OTUD7B and VEGF expression is still unclear. An IHC analysis of 171 NSCLC patient specimens revealed that VEGF was significantly upregulated in cancer tissues compared with adjacent tissues. Patients whose NSCLC featured high OTUD7B expression had high a VEGF level and especially poor prognosis. Further studies in NCI-H358 cells suggested that OTUD7B promoted VEGF secretion and facilitated endothelial cell tube formation *in vitro*. In addition, OTUD7B was demonstrated to promote tumor growth in NCI-H358 lung cancer xenografts. These findings may have significant implicants for the treatment of NSCLC via targeting OTUD7B.

Taken together, this study identified OTUD7B as an independent predictive factor for the prognosis of LUSC and LAD. OTUD7B promotes lung cancer cell proliferation and migration via Akt/VEGF signal pathway. Strategies to silencing OTUD7B in carcinoma tissues may inform the development of novel therapies to fight against LUSC and LAD.

## Data Availability

The datasets generated for this study are available on request to the corresponding author.

## Ethics Statement

This study was carried out in accordance with the recommendations of Institutional Review Board of Hebei University Affiliated Hospital with written informed consent from all subjects. All subjects gave written informed consent in accordance with the Declaration of Helsinki. The protocol was approved by the Institutional Review Board of Hebei University Affiliated Hospital. This study was carried out in accordance with the recommendations of the Animal Research Ethics Committee of Hebei University. The protocol was approved by the Animal Research Ethics Committee of Hebei University.

## Author Contributions

DL, YS, SQ, WL, LZ, NC, and YuW performed the experiments. DL, YS, YaW, and NC collected and analyzed data. SZ and JS conceived and designed the project. DL, SZ, and JS wrote the manuscript.

### Conflict of Interest Statement

The authors declare that the research was conducted in the absence of any commercial or financial relationships that could be construed as a potential conflict of interest.
