# Supplementary material for: Upregulation of OTUD7B (Cezanne) Promotes Tumor Progression via AKT/VEGF Pathway in Lung Squamous Carcinoma and Adenocarcinoma
Source: Front Oncol. 2019 Sep 11;9:862. doi: 10.3389/fonc.2019.00862 (PMC6749047; doi:10.3389/fonc.2019.00862)
Supplement: Supplementary file 1 [file Data_Sheet_1.pdf]

Supplementary Material

Supplementary Figures

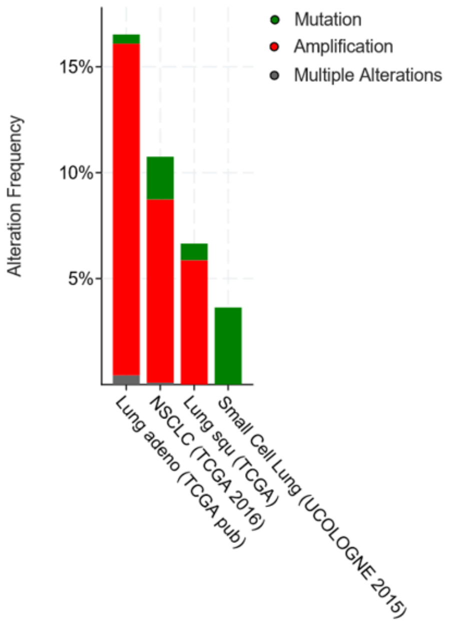

Supplementary Figure S1. TCGA DNA sequencing of OTUD7B gene in lung cancer. TCGA DNA sequencing results showing that OTUD7B gene is amplified at high frequencies in NSCLC, including LUAD and LUSC.

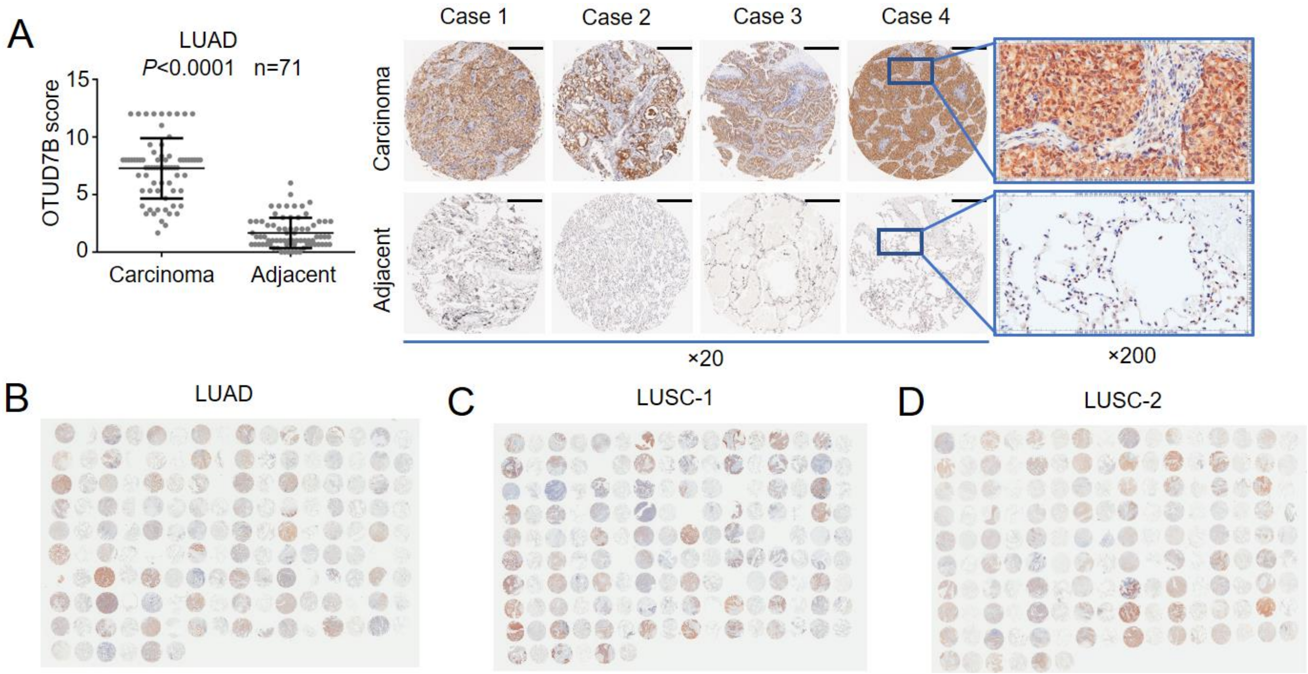

Supplementary Figure S2. Elevated OTUD7B expression in LUAD is associated with cancer progression. (A) OTUD7B expression scores in lung adenocarcinoma are shown. Lung adenocarcinoma tissues were compared with matched adjacent normal tissues using paired *t* test. Representative images of IHC staining of OTUD7B expression from 4 cases are shown. Magnification, ×20 (left) and ×200 (right); Scale bars, 400 μm. **(B-D)** The LUAD **(B)** and LUSC **(C and D)** microarray IHC staining with anti-OTUD7B antibody.

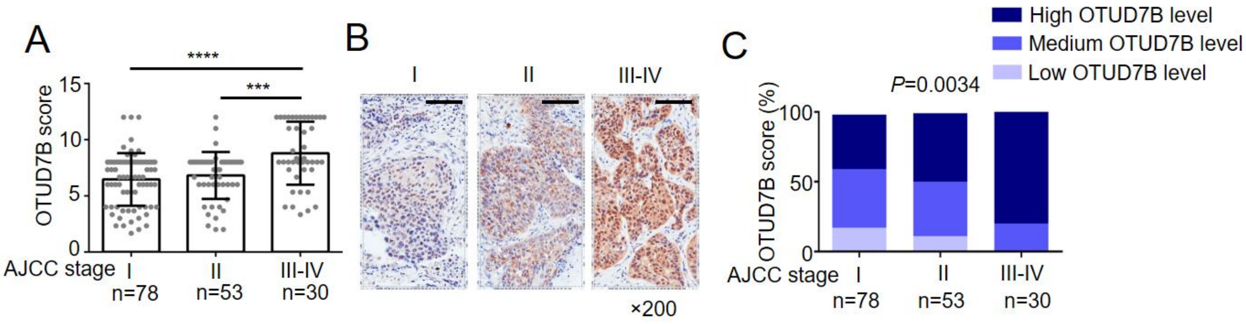

Supplementary Figure S3. OTUD7B upregulation is associated with AJCC stage in NSCLC. **(A)** OTUD7B score in tumors with different AJCC stages (AJCC 7th Edition Clinical Staging). Data were analyzed using one-way ANOVA and Tukey's multiple comparisons test and are shown as mean ± s.d. Each dot represents an individual sample. ***,P<0.001, ****, P<0.0001. **(B)** Representative images from IHC staining of OTUD7B in different AJCC stages are shown. Magnification, ×200; scale bars, 100 μm. **(C)** The percentage of tumours with different AJCC stages. Data were analyzed using Pearson's χ^2 test.

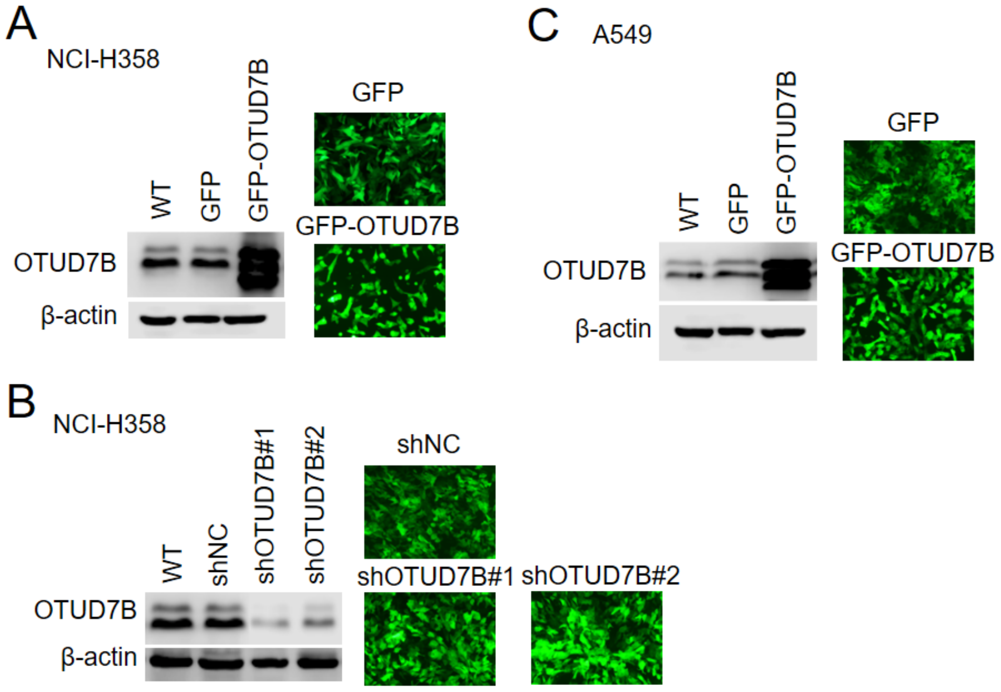

Supplementary Figure S4. OTUD7B overexpression and knockdown in NSCLC cell lines. (A) NCI-H358 cells were transduced with lentivirus expression vectors to get GFP or GFP-OTUD7B overexpression cells. Total cell lysates were subjected to immunoblotting using anti-OTUD7B antibody. GFP and GFP-OTUD7B expression efficiency were visualized using fluorescence microscope (*right panel*). WT, wild type. **(B)** NCI-H358 cells were transduced with a non-targeting control shRNA (shNC) or two different OTUD7B-specific shRNAs. Total cell lysates were subjected to immunoblotting using anti-OTUD7B antibody. Transduce efficiency were visualized using fluorescence microscope (*right panel*). **(C)** A549 cells were transduced with lentivirus expression vectors to get GFP or GFP-OTUD7B overexpression cells. Total cell lysates were subjected to immunoblotting using anti-OTUD7B antibody. GFP and GFP-OTUD7B expression efficiency were visualized using fluorescence microscope (*right panel*). WT, wild type.

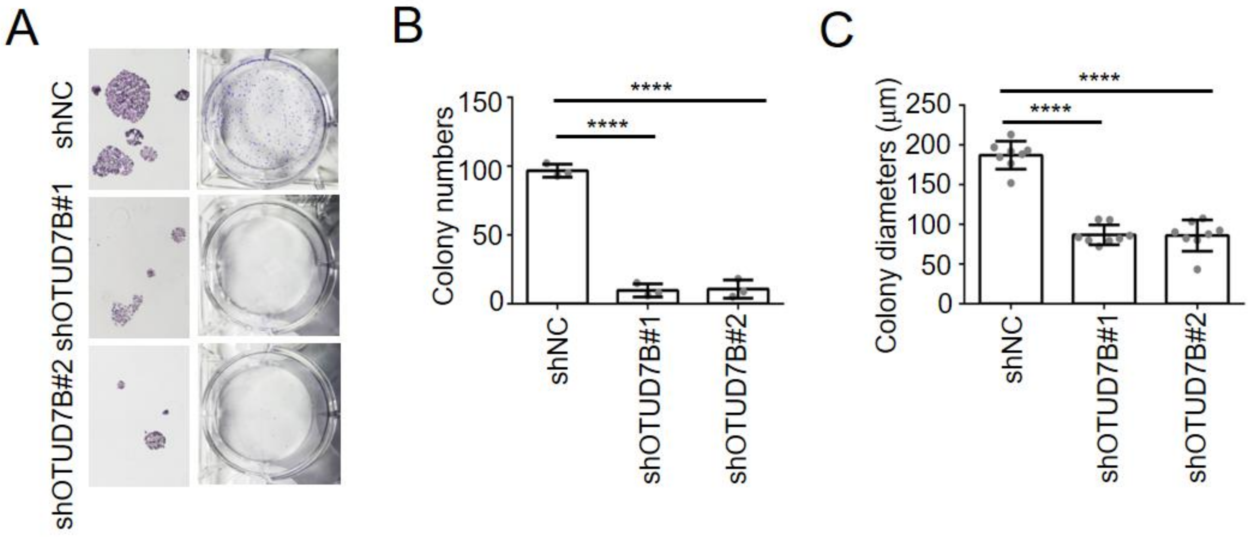

Supplementary Figure S5. Knockdown of OTUD7B reduces proliferation in NCI-H358 cells. NCI-H358 cells were transduced with shNC or shOTUD7B#1 or #2 and colony formation was preformed to determine cell proliferation. (A) Representative images. (B) Colony numbers in each group. (C) Colony diameters in each group. Data are shown as mean ±s.d. ****, $P < 0.0001$.

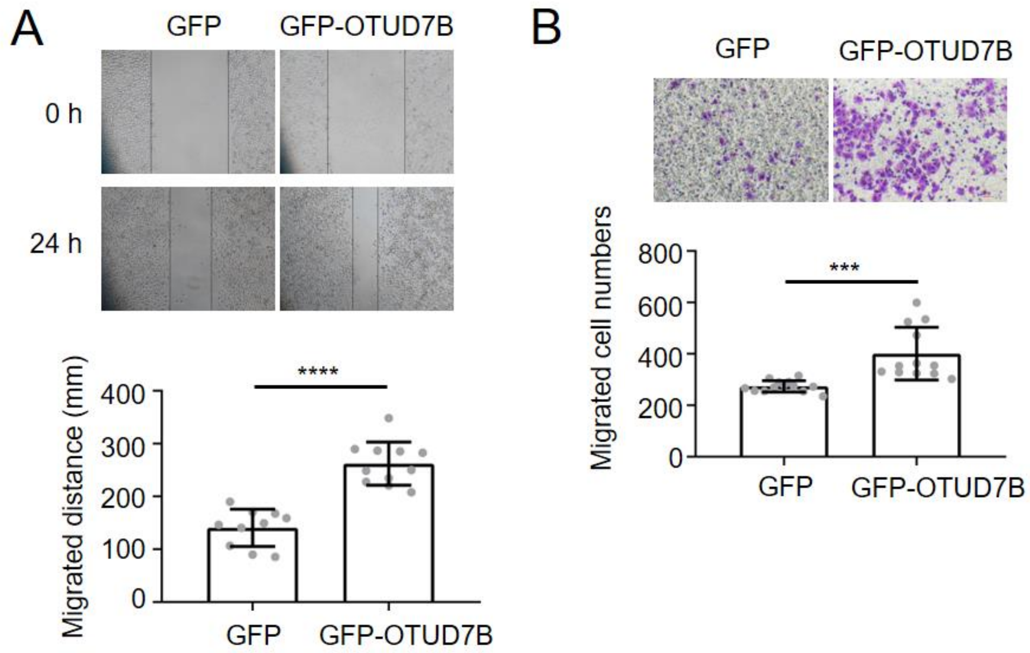

Supplementary Figure S6. OTUD7B enhances migration and invasion in A549 cells. A549 lung cancer cells were transduced with GFP or GFP-OTUD7B. Wound healing assay **(A)** and transwell invasion assay using Boyden chamber **(B)** in A549 cells were performed and photographed under a light microscope (magnification, ×100). Representative images are shown (*upper panels*). Data are shown as mean ±s.d. ***, $P<0.001$, ****, $P < 0.0001$.

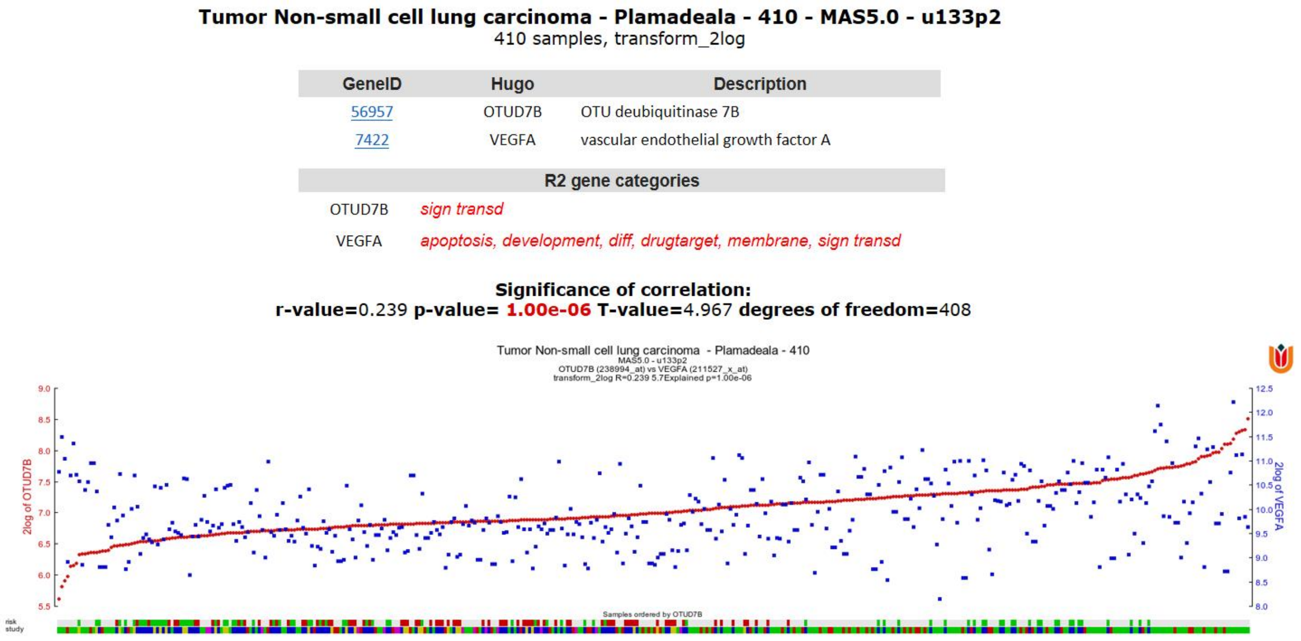

Supplementary Figure S7. Correlation analysis between OTUD7B and VEGF using R2: Genomics Analysis and Visualization Platform system. Tumor Non-small cell lung carcinoma-Plamadeala-410-MAS5.0-u133p2 dataset included 410 NSCLC patients was selected to observe the expression correlation between OTUD7B and VEGF.

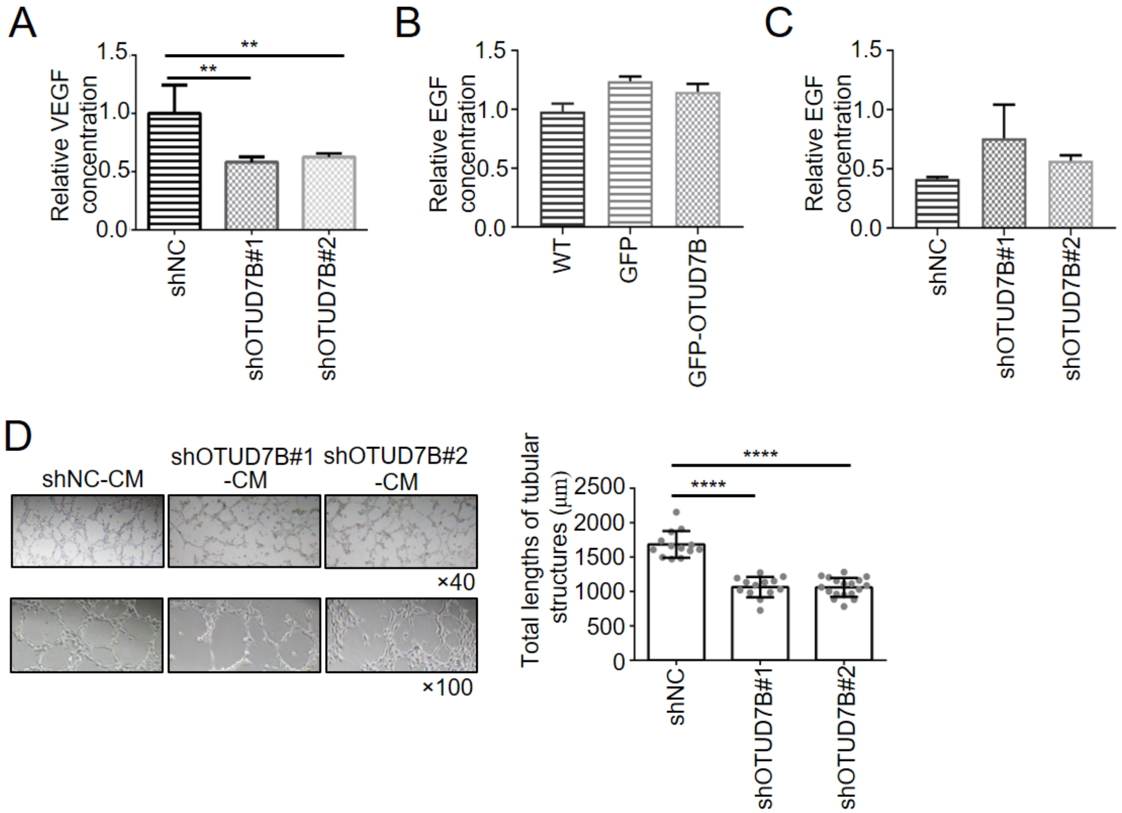

Supplementary Figure S8. Knockdown of OTUD7B decreases VEGF production in NCI-H358 cells. (A) NCI-H358 cells were transduced with shNT, shOTUD7B#1 or shOTUD7B#2, media was removed, washed in RPMI-1640 with 0.5% FBS and incubated for an additional 24 h in RPMI-1640 with 0.5% FBS. Conditioned media were collected and VEGF expresseion was analyzed by ELISA assay. **(B)** NCI-H358 cells were transduced with GFP or GFP-OTUD7B expression vector, conditioned media were collected and EGF expression was analyzed by ELISA assay. **(C)** Conditioned media from NCI-H358 cells were collected and EGF expression were analyzed by ELISA assay. **(D)** EA.hy926 endothelial cells were pretreated with conditioned media collected from NCI-H358 cells (shNT-CM, shOTUD7B#1-CM or shOTUD7B#2-CM) for 24 h. Subsequently, pretreated EA.hy926 cells were seeded on Matrigel for 8 h to observe tube formation. Representative photographs are shown (left). Tube lengths were quantitated using IMAGE-PRO PLUS software (n = 5 per group). Data are shown as mean ±s.d. ***, P<0.001, ****, P<0.0001.

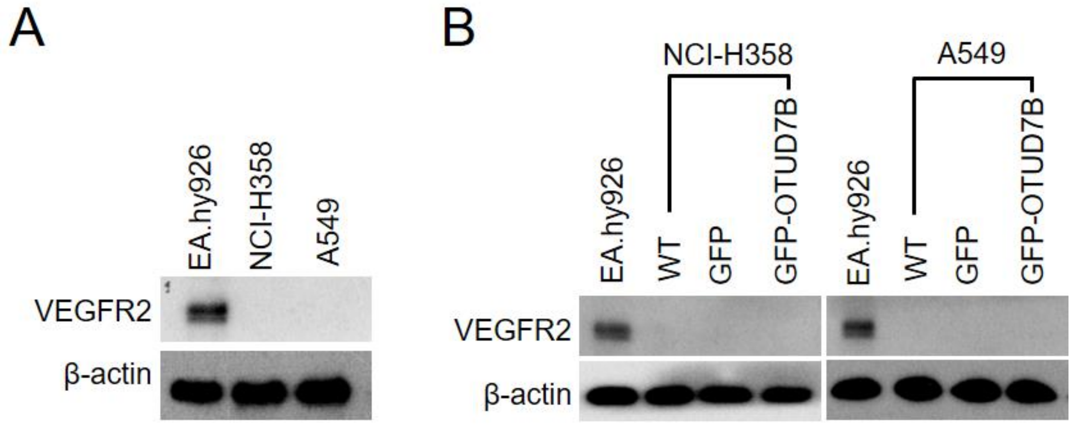

Supplementary Figure S9. VEGFR2 expression in EA.hy926 endothelial cells and NSCLC cell lines. (A, B) EA.hy926 endothelial cells, NCI-H358 and A549 NSCLC cells transduced with GFP or GFP-OTUD7B or wild type (WT) cells were lysed and total cell lysates were subjected to immunoblotting assay using indicated antibodies.

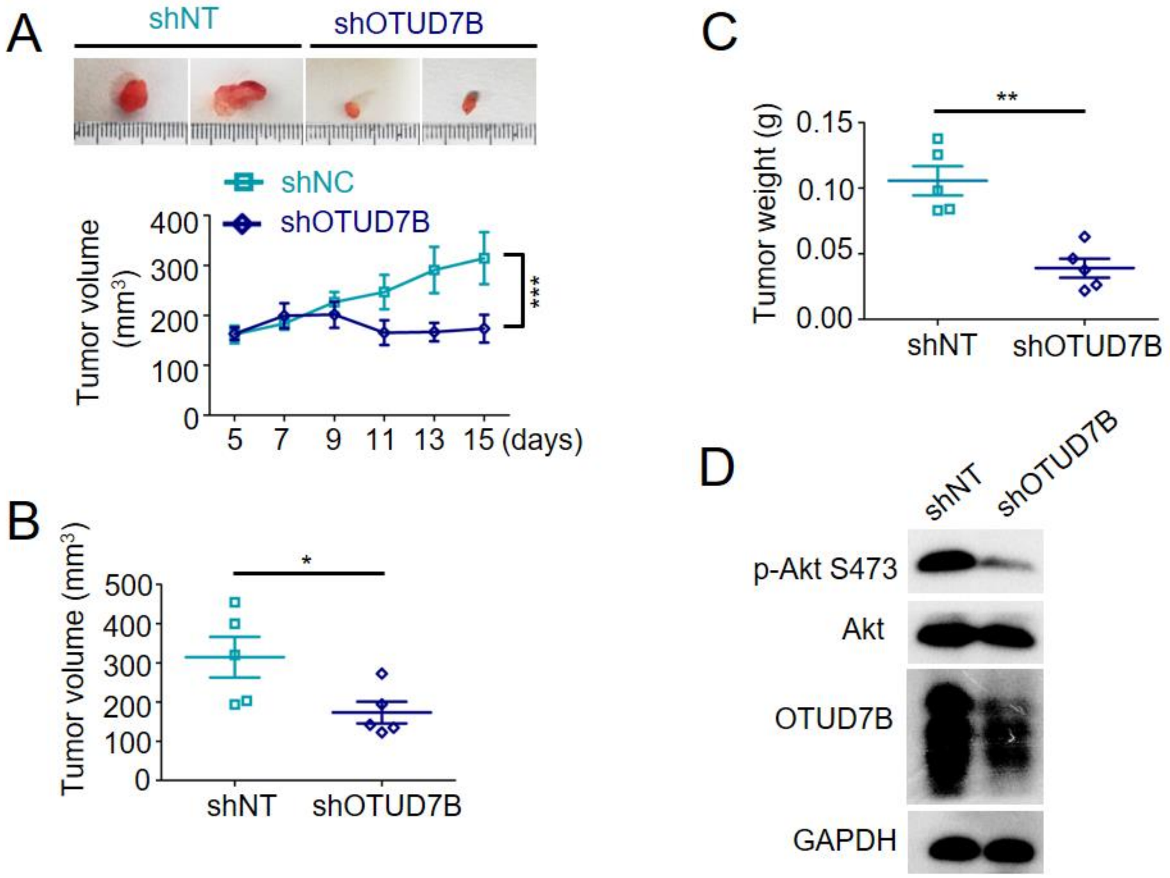

Supplementary Figure S10. Knockdown of OTUD7B reduces *in vivo* tumorigenicity of NSCLC. **(A)** Nude mice were injected s.c. with shNT- or shOTUD7B-NCI-H358 cells at 5×10^6 cells per site. Tumor volume was monitored every 2 days after subcutaneous injection and tumor growth curve is shown (lower). Tumor volume was calculated by the formula: $V=1/2\times a$ (length)$\times b^2$ (width). Tumors were harvested from the mice 15 days after subcutaneous injection and photographed (upper). (n=5 per group). **(B and C)** The volume (B) and the weight (C) of the harvested tumors was measured. (D) Total lysates of tumor tissues were subjected to immunoblotting using anti-p-Akt and anti-Akt antibodies. Data are shown as mean ±s.e.m. *, *P*<0.05, **, *P*<0.01, ***, *P*<0.001.
